# A New Coding System for the Identification of Left Ventricular Rotation Patterns and Their Relevance to Myocardial Function

**DOI:** 10.1007/s10439-024-03539-4

**Published:** 2024-06-09

**Authors:** Vicente Mora, Juan Geraldo, Ildefonso Roldán, Ester Galiana, Celia Gil, Pablo Escribano, Rosina Arbucci, Alberto Hidalgo, Paula Gramage, Jorge Trainini, Francesc Carreras, Jorge Lowenstein

**Affiliations:** 1 Department of Cardiology, Hospital Universitario Dr Peset, 46017 Valencia, Spain; 2 Cardiodiagnosis Department, Medical Research, 1425 Buenos Aires, Argentina; 3 Department of Cardiology, Hospital Sant Pau, 08025 Barcelona, Spain; 4 Cardiology Department, Universitat de València, Hospital Universitario Dr Peset, Avda Gaspar Aguilar 90, 46017 Valencia, Spain

**Keywords:** Speckle tracking echocardiography, Twist, Rigid rotation, Rotational gradient, Myocardial function, Left ventricular ejection fraction

## Abstract

Rotational mechanics is a fundamental determinant of left ventricular ejection fraction (LVEF). The coding system currently employed in clinical practice does not distinguish between rotational patterns. We propose an alternative coding system that makes possible to identify the rotational pattern of the LV and relate it to myocardial function. Echocardiographic images were used to generate speckle tracking-derived transmural global longitudinal strain (tGLS) and rotational parameters. The existence of twist (basal and apical rotations in opposite directions) is expressed as a rotational gradient with a positive value that is the sum of the basal and apical rotation angles. Conversely, when there is rigid rotation (basal and apical rotations in the same direction) the resulting gradient is assigned a negative value that is the subtraction between the two rotation angles. The rotational patterns were evaluated in 87 healthy subjects and 248 patients with LV hypertrophy (LVH) and contrasted with their myocardial function. Our approach allowed us to distinguish between the different rotational patterns. Twist pattern was present in healthy controls and 104 patients with LVH and normal myocardial function (tGLS ≥ 17%, both). Among 144 patients with LVH and myocardial dysfunction (tGLS < 17%), twist was detected in 83.3% and rigid rotation in 16.7%. LVEF was < 50% in 34.7%, and all patients with rigid rotation had a LVEF < 50%. The gradient rotational values showed a close relationship with LVEF (*r* = 0.73; *p* < 0.001). The proposed coding system allows us to identify the rotational patterns of the LV and to relate their values with LVEF.

## Introduction

Since Leonardo da Vinci and Richard Lower [1] described the helical structure and rotational movement of the human heart in the sixteenth and seventeenth centuries, respectively, the study of the mechanics of this organ has continued. Today, the close relationship between ventricular structure and its correct function is widely recognized.

The oblique orientation of the myocardial fibers along the wall of the heart determines the main direction of the mechanical force produced by the shortening of the cardiomyocytes. In the left ventricular (LV) wall, this oblique arrangement of the myofibers changes progressively as a function of depth [2–4] and follows a helical path around the ventricular cavity. As a rule, the endocardial fibers adopt a positive helical angle of approximately + 60° and progressively change their orientation at the transmural level, eventually adopting a negative angle of approximately − 60°. This implies that cardiomyocyte shortening occurs longitudinally and circumferentially, with the fibers adopting different orientations depending on their transmural location. This interaction between longitudinal and circumferential contraction dictates the nature of ventricular rotation. If the normal adult heart is viewed from the apex, what is perceived as rotation of the base in a clockwise direction and rotation of the apex in a counterclockwise direction generate a “twist” motion. Simultaneously with these rotational movements, the LV shortens longitudinally as the base moves toward the apex [5–7]. Conversely, if the base and apex rotate in the same direction, there is no twisting motion, a situation described as “rigid rotation” [8]. In the current coding system, counterclockwise rotation is expressed in positive values, while rotation in the clockwise direction is expressed in negative values. The rotational gradient is the result of the interaction between the basal and apical rotations of the LV.

In the past, the evaluation of LV rotational mechanics required invasive methods [9–11], but since the beginning of the twenty-first century non-invasive imaging techniques have been available for this purpose, with cardiac resonance representing the gold standard. Indeed, the incorporation of speckle tracking echocardiography (STE) into clinical practice has revived the study of LV rotational mechanics, which have been shown to correlate closely with cardiac resonance (*r* = 0.93) [12–17].

Different rotational patterns -namely twist and rigid rotation- have been identified in several heart diseases in which LVEF is affected. However, the coding system that is currently incorporated into echocardiography devices wrongly assumes that all rotational models in the LV indicate the existence of twist. Thus, despite the relevance of rotational mechanics in LVEF, the way in which they are currently codified does not allow them to be distinguished from other rotational models in a reliable way. This probably explains why the literature on rotational mechanics and LVEF is scarce.

We propose a new approach to codification that makes it possible to qualitatively identify the LV rotational pattern and to quantitatively relate rotational gradient values to LVEF.

## Methods

### Study Population

We applied our system prospectively to 335 subjects; first to 87 healthy subjects and subsequently to 248 consecutive patients with LV hypertrophy (LVH). Patients with prosthetic valves (4), pacemakers (5), and cardiac resynchronization devices (2) were excluded, as were those with a deficient acoustic window (19). Patients with hypertrophic myocardiopathy (37) were also excluded due to their aberrant myocardial distribution.

The study complied with the principles of the Helsinki Declaration of 1975 and was approved by the ethics committee of both institutions. All patients gave their informed consent.

### Echocardiography

Two ultrasound systems (Vivid E9 and Vivid E95; GE Healthcare, Little Chalfont, United Kingdom), each equipped with a 2.5-MHz transducer, were employed in the study population to obtain 2D echocardiographic images from the parasternal short axis view at the basal, mid, and apical levels and from the 3 standard LV apical view (4-, 2-, and 3-chambers). These images were used to calculate speckle tracking-derived rotational parameters, transmural global longitudinal strain (tGLS) and transmural global circumferential strain (tGCS).

Two-dimensional echocardiographic images confirmed interventricular septal thickness > 12 mm in all the patients with LVH as inclusion criteria. Images of four and two chambers were recorded for analysis of end-diastolic and end-systolic volumes. LVEF was calculated using the Simpson biplane method.

**Fig. 1:**
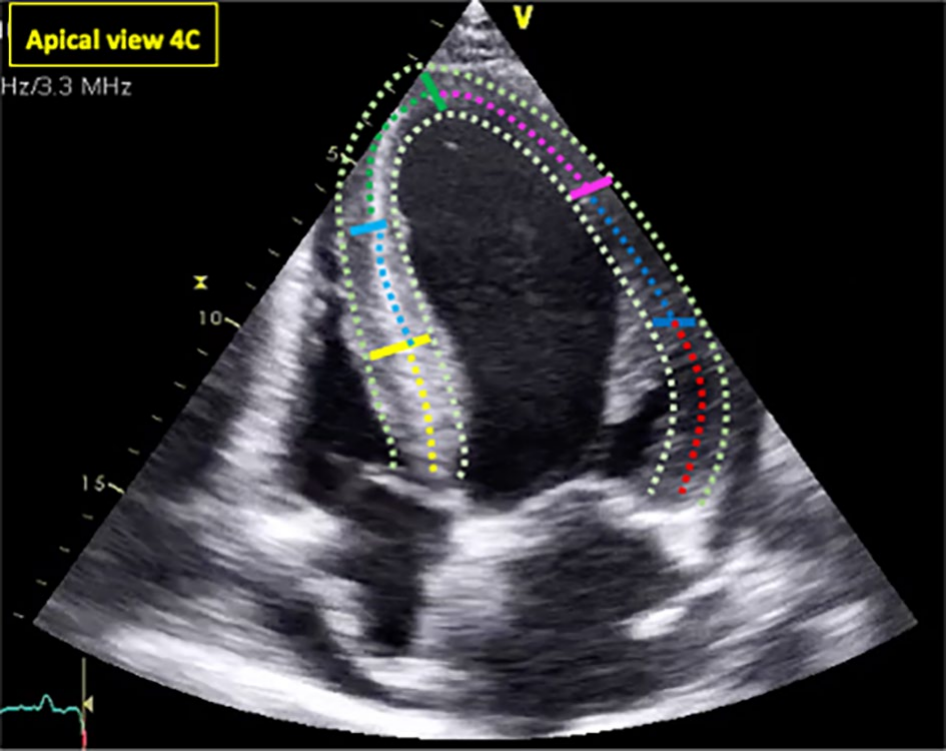
To determine strain, the endocardial edge of the ventricle is traced just inside the myocardium. A second wider concentric circle is automatically generated next to the epicardium, so as to include the entire transmural thickness (verified by the operator) of the myocardial wall

To determine strain, the endocardial edge of the ventricle is traced just inside the myocardium. With the help of software, a second wider concentric circle is automatically generated next to the epicardium, so as to include the entire transmural thickness of the myocardial wall (Fig. 1). The basal plane is traced at the middle level of the mitral valve using its opening during diastole as a reference, and the apical plane is traced below the insertion of the papillary muscles, avoiding visualization of the right ventricle as much as possible. The program divides each projection into six equal segments and performs frame-by-frame speckle tracking of the myocardium, providing automatized tracking confirmation (verified by the operator) and generating strain values expressed as percentages (%) of shortening, while angular displacements are expressed in degrees (°).

We have expressed tGLS and tGCS in positive values to facilitate understanding. tGLS < 17% was considered to represent myocardial dysfunction. All images were obtained at a rate of 50–80 frames/s and transferred to a workstation for computer analysis (EchoPAC version 203; GE Healthcare, Little Chalfont, United Kingdom).

The term rotation refers to the angular displacement (in degrees) of a myocardial segment in a transversal projection around the longitudinal axis of the LV, either clockwise or counterclockwise direction (this is how it would appear when viewed from the apex) [13].

“Twist” (also referred to as torsion if twist is measured with respect to the longitudinal diameter of the LV) occurs when the base and apex rotate in opposite directions. If they rotate in the same direction the movement is described as “rigid rotation”, in which case there is no twist.

**Fig. 2:**
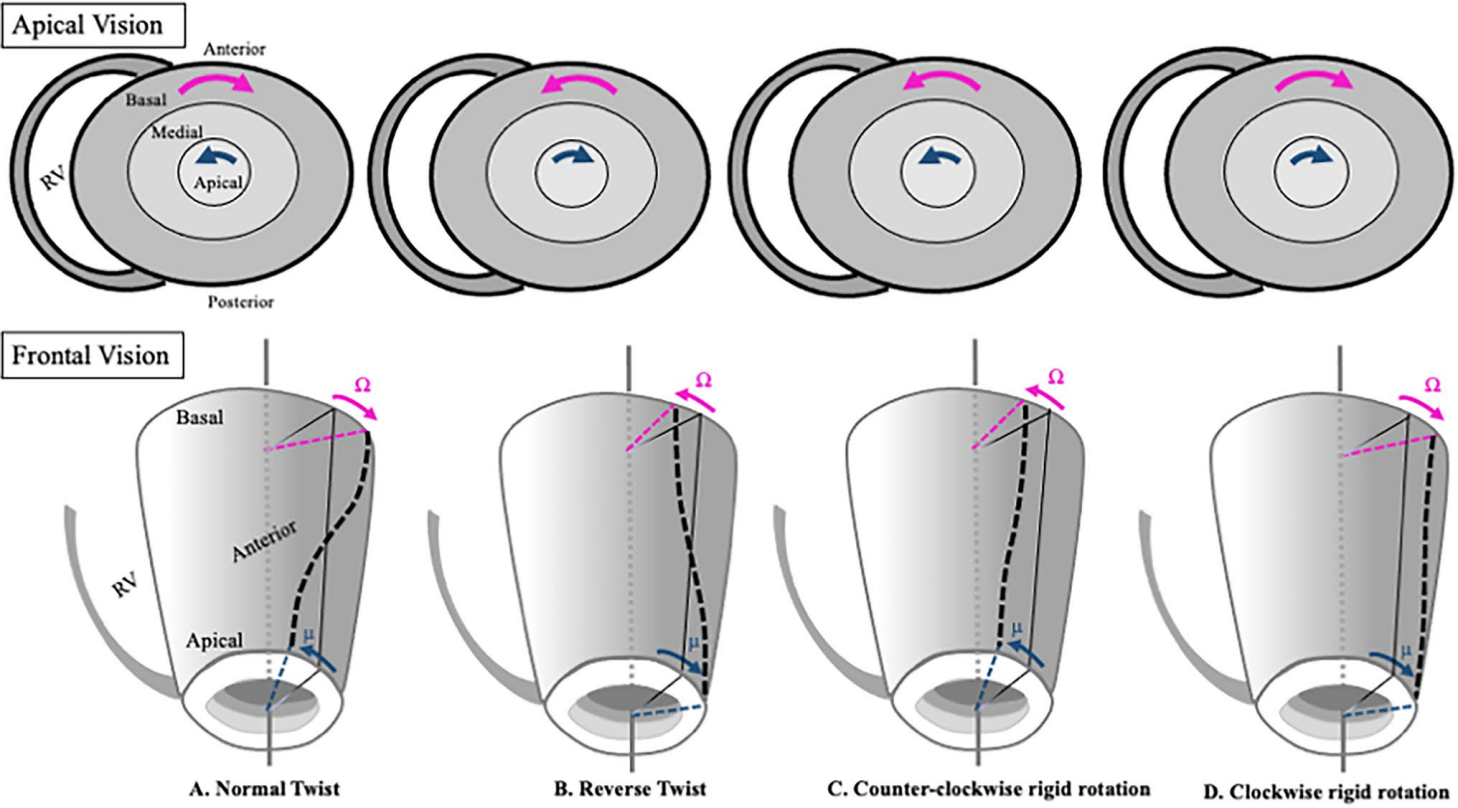
Rotational models in the left ventricle. Normal (A), reverse (B), counter-clockwise rigid rotation (C) and clockwise rigid rotation (D)

There are several models of left ventricle rotation (Fig. 2A–D):

A. Normal twist: basal rotation occurs in a clockwise direction and apical rotation in a counterclockwise direction (Fig. 2A).
B. Reverse twist: unlike normal twist, basal rotation occurs in a counterclockwise direction and apical rotation in a clockwise direction (Fig. 2B).
C. Clockwise rigid rotation: Basal and apical rotation both occur in a clockwise direction (Fig. 2C).
D. Counterclockwise rigid rotation: basal and apical rotation both occur in a counterclockwise direction (Fig. 2D).
E. Semi-rigid rotation: term coined to define a mixed behavior outside the isometric contraction period of systole, in which systolic rotation involves both twisting movement and rigid rotation.

### Coding System

**Fig. 3:**
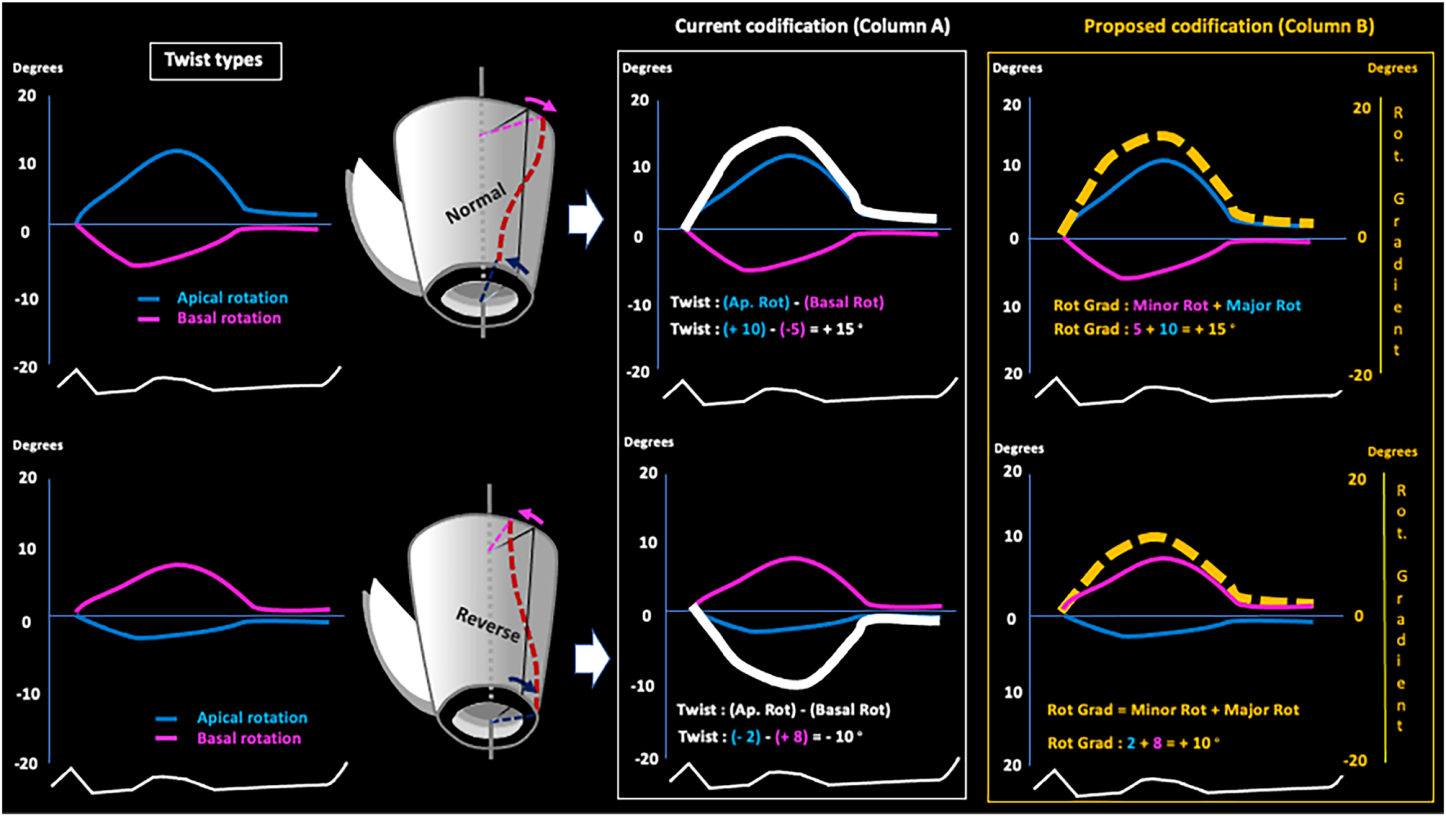
Twist rotational pattern. Schematic current (column A, white line) and alternative (column B, broken yellow line) systems for codifying twist types of LV rotational pattern. See text. *Ap. rot*: apical rotation, *Basal rot*: basal rotation, *Rot. Gradient*: rotational gradient

**Fig. 4:**
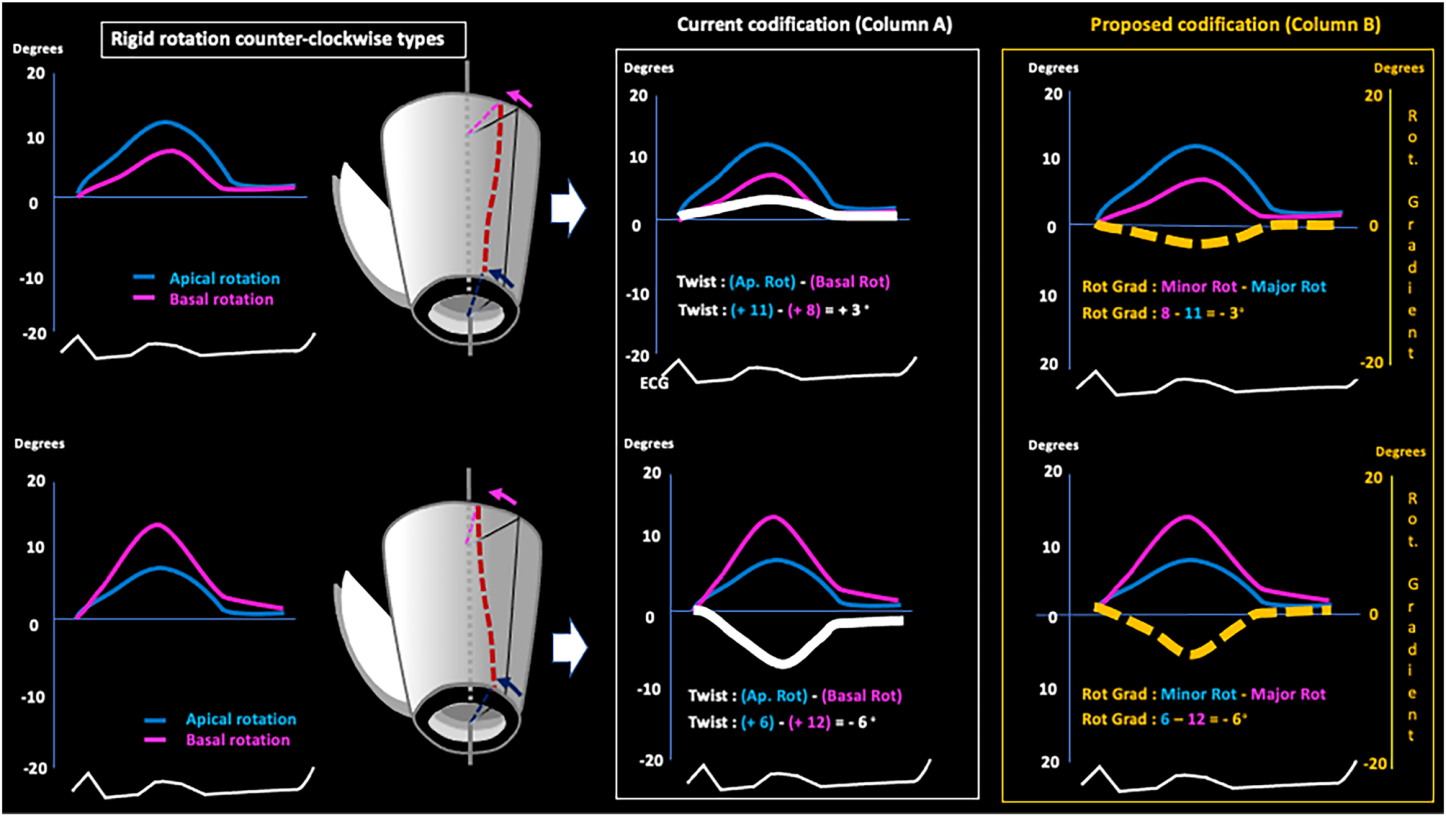
Counterclockwise rigid rotational pattern. Schematic current (column A, white line) and alternative (column B, broken yellow line) systems for codifying counterclockwise rigid rotation types LV rotational pattern. See text. *Ap. rot* apical rotation, *Basal rot* basal rotation, *Rot. Gradient* rotational gradient

**Fig. 5:**
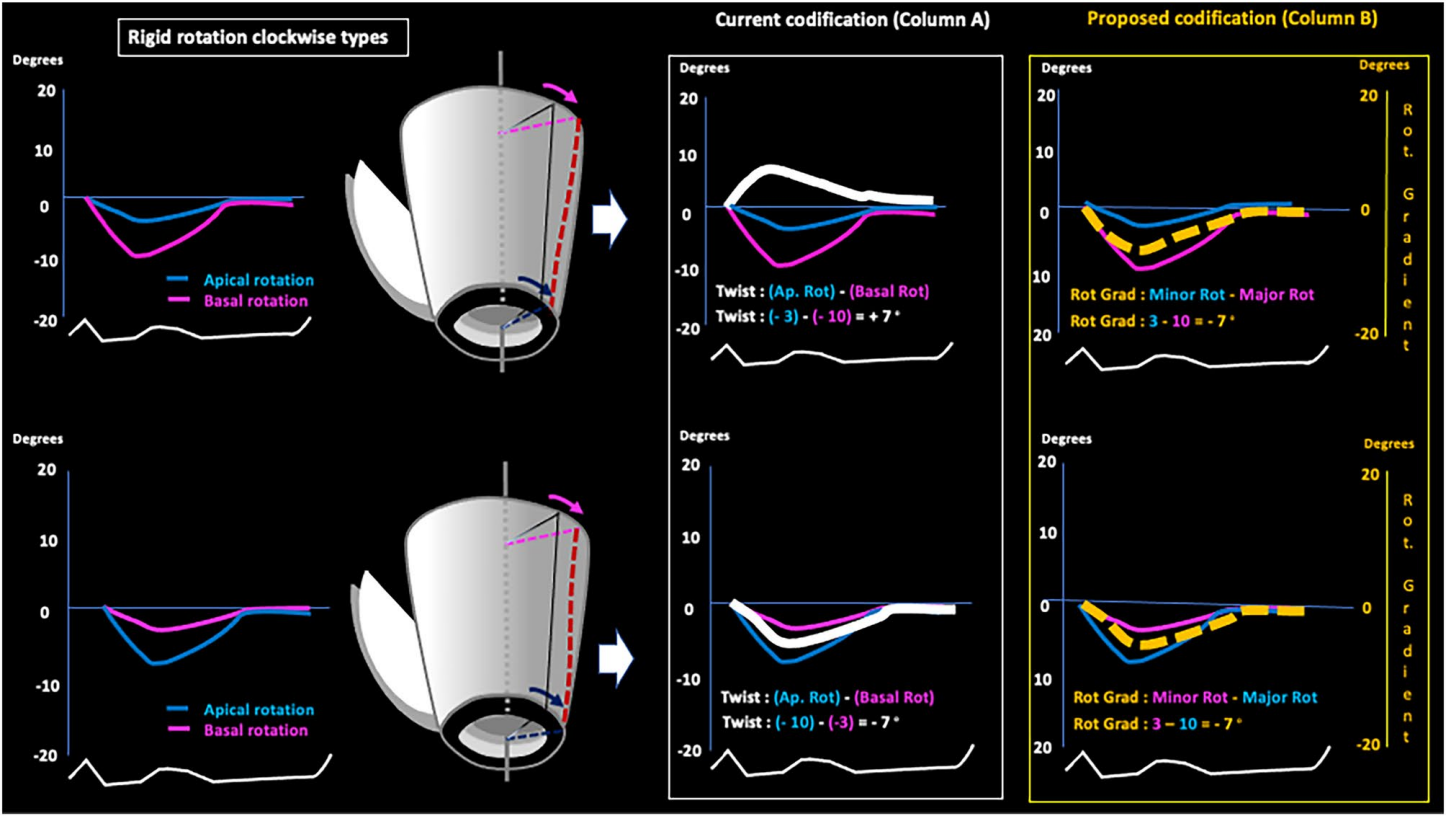
Clockwise rigid rotational pattern. Schematic current (column A, white line) and alternative (column B, broken yellow line) systems for codifying clockwise rigid rotation types LV rotational pattern. See text. *Ap. rot* apical rotation, *Basal rot* basal rotation, *Rot. Gradient* rotational gradient

The currently employed coding system wrongly assumes that all rotational models in the LV indicate the existence of twist, calculated through algebraic subtraction by the formula Twist° = (apical rotation)° − (basal rotation)°. Thus, in contrary situations like the presence versus absence of twist (rigid rotation, in the latter case) the resulting rotational gradient can be coded indistinctly as positive or negative, which does not allow them to be differentiated (Figs. 3, 4, 5 column A, white line).

In order to identify and differentiate these rotational patterns, we propose a new approach to coding in which basal and apical rotation angles are represented in absolute values, without signs of positivity or negativity that can be interpreted, respectively, as beneficial or deleterious, when in fact they only indicate the direction of the rotation (clockwise vs counterclockwise in both). The rotational gradient is calculated according to the following Algorithm 1:

$$^{*}{\text{When there is twist}},\,{\text{Rotational gradient}}^{\circ} = {\text{Minor rotation}}^{\circ} + {\text{Major rotation}}^{\circ}$$

$$^{*}{\text{When there is rigid rotation}},\,{\text{Rotational gradient}}^{\circ} = {\text{Minor rotation}}^{\circ} - {\text{Major rotation}}^{\circ}$$

Thus, rotational gradient is calculated with a simple algorithm that can be easily incorporated into currently available echocardiography equipment (and to any cardiac imaging modality), in which the existence of twist (basal and apical rotations in opposite directions) is always expressed as a rotational gradient with a positive value that is the sum of the basal and apical rotation angles (Fig. 3, column B, broken yellow line). Conversely, when there is rigid rotation (basal and apical rotations in the same direction) the resulting gradient is always assigned a negative value that is the subtraction between the two rotation angles (Figs. 4 and 5, column B, broken yellow line).

It is important to emphasize that the direction of apical or basal “rotation” (clockwise vs counterclockwise) should not be confused with the “rotational gradient” (twist-positive vs rigid rotation-negative).

Having established our new coding system, it was important to assess its relevance in clinical practice. In order to this we determined the relationship of the different rotational models with myocardial function (normal if tGLS $\ge$ 17%) and LVEF (normal if LVEF $\ge$ 50%).

### Statistical Analysis

Continuous variables are represented by the mean and standard deviation (SD), and proportions are shown as percentages. Analysis of variance (ANOVA) was employed to compare groups, while the Tukey honestly significant difference (HSD) test was used to discern post hoc differences between pairs of means and the Chi-square test was employed for categorical variables. The relationship between LVEF (dependent variable) and rotational gradients (independent variable) was estimated using linear regression. The *R* values of the bivariate correlations originated from the Pearson Correlation Coefficient. The Coefficient is provided with the 95% confidence intervals. To control the clustering effect, the *R* values and the 95% confidence interval of the relationship between LVEF and the rotational gradient were obtained by applying the bias adjustment of the estimation of the confidence limits. *P* values < 0.05 were considered to indicate statistical significance in all the analyses. SPSS Statistics for Windows version 26.0 (IBM, Armonk, NY) and MedCalc Statistical Software version 20.014 (MedCalc Software, Ostend, Belgium) were employed for statistical analyses and graphs.

## Results

### Coding Types

**Fig. 6:**
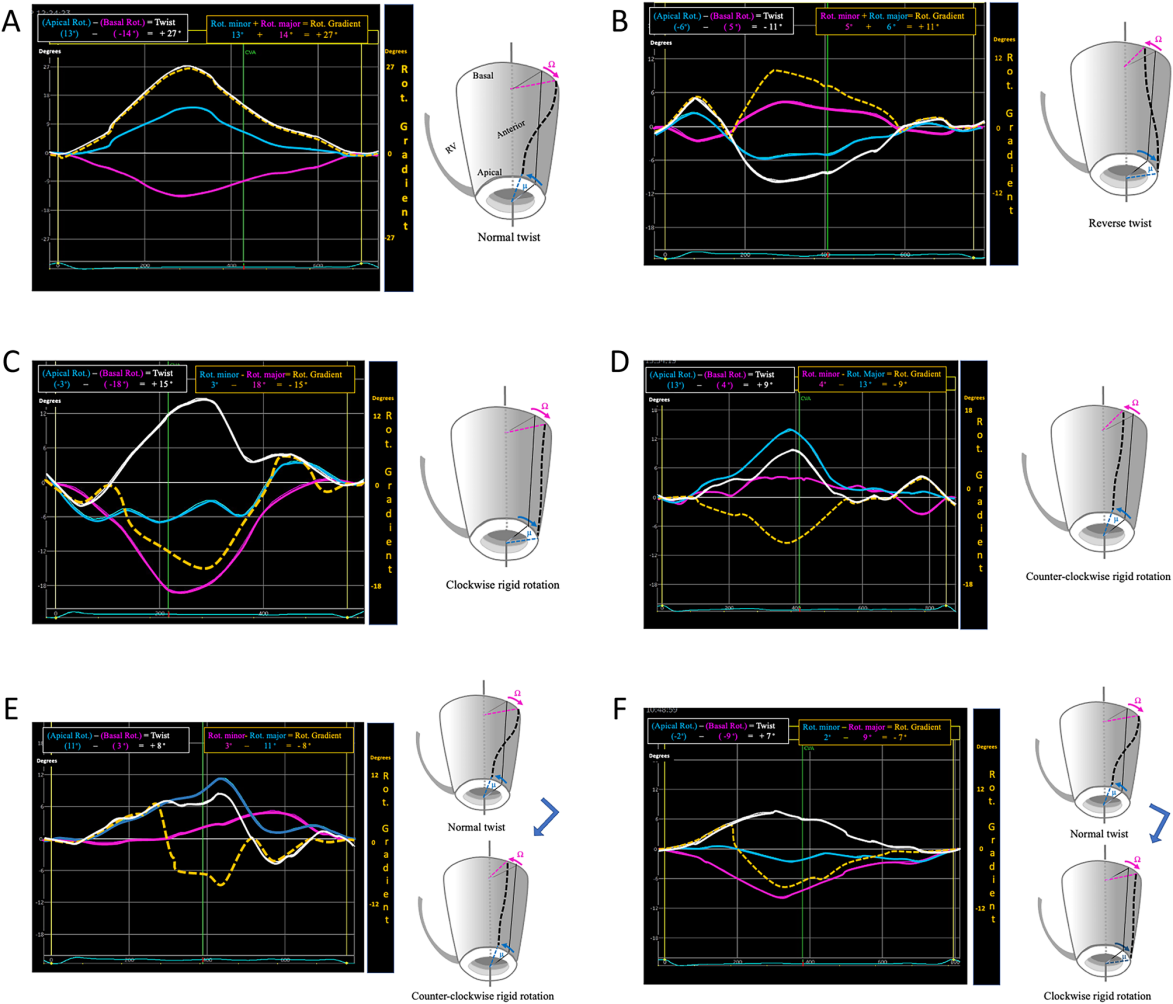
Examples of different rotation models obtained in our study population. Representation according to coding system. With the currently used coding system the curve of rotational gradient is represented as a continuous white line when visualized through an echocardiography device. With the coding system we propose, the curve of rotational gradient is represented as a broken yellow line (manual layout), and presents always as positive if there is twist and negative if there is rigid or semi-rigid rotation. Normal (**A**) and reverse (**B**) twist, which there is apical and basal rotation in opposite directions. Counter-clockwise (**C**) and clockwise (**D**) rigid rotation, which is produced when there is apical and basal rotation in the same direction (or absence of twist). Counter-clockwise (**E**) and clockwise (**F**) semi-rigid rotation, in which the apex and base initially rotate in opposite directions and eventually rotate in the same direction

Figure 6 shows the types of twist (Fig. 6A, B), rigid rotation (Fig. 6C, D) and semi-rigid rotation (Fig. 6E, F) identified. With the current coding system (white line), the rotational gradient in twist and rigid rotation can appear indistinctly as a positive or negative value. With the system we propose (broken yellow line), the rotational gradient between basal and apical rotation will be a positive value when there is twist (normal or reverse), while the rotational gradient between basal and apical rotation will be a negative value when there is rigid or semi-rigid rotation, regardless of whether it is clockwise or counterclockwise.

### Coding System, Myocardial Function and LVEF

Three hundred and thirty-five subjects − 87 healthy controls and 248 patients with LVH- met all the inclusion criteria and none of the exclusion criteria. The cardiopathies underlying LVH in each case were as follows: 105 patients had aortic stenosis, 51 had amyloidosis, 5 had aortic stenosis and amyloidosis, and 87 had hypertensive cardiomyopathy. The results of the analysis between groups are summarized in Table 1.

**Table 1:** Characteristics of healthy controls and patients

	Controls (*n* = 87)	LVH (*n* = 248)		*p*
	tGLS $\ge$ 17% (*n* = 87) G-1	tGLS $\ge$ 17% (*n* = 104) G-2	tGLS < 17% (*n* = 144) G-3	
Age, years	53.3 ± 13.4	70.7 ± 12.9^a^	74,0 ± 14.8^b^	< 0.001
Male (%)	42.6%	50.9%	72.2%	< 0.001
HR beats/min	65.1 ± 10.6	68.6 ± 11.6	75.0 ± 13.7^b,c^	< 0.001
sBP, mmHg	118.5 ± 16.5	150.0 ± 19.3^a^	146.2 ± 32.9^b^	< 0.001
LVEDV, ml	89.7 ± 28.8	74.2 ± 24.4^aa^	80.7 ± 36.0^bb^	< 0.01
LVESV, ml	30.1 ± 10.3	23.6 ± 9.7^a^	39.0 ± 26.8^b,c^	< 0.001
LVEF, %	66.6 ± 5.52	68.5 ± 6.09	53.8 ± 14.5^b,c^	< 0.001
LVEF < 50%	0 (0%)	0 (0%)	50 (34,7%)	
tGLS, %	21.4 ± 1.94	20.4 ± 2.07	11.7 ± 3.48^b,c^	< 0.001
tGCS, %	22.2 ± 4.7	22.1 ± 3.7	15,9 ± 4.2^bb,cc^	< 0.01
Twist, °	19.9 ± 7.5	26.8 ± 11.2^a^	16.1 ± 13.8^b,c^	< 0.001

Continuous variables are represented by the mean ± DE, and proportions are shown as percentages. Analysis of variance (ANOVA) was employed to compare groups, and the Tukey honestly significant difference (HSD) test was used to discern post hoc differences between pairs of means and the Chi-square test for categorical variables

*tGLS* transmural global longitudinal strain, *tGCS* transmural global circumferential strain, *LVH* left ventricular hypertrophy, *HR* heart rate, *LVEF* left ventricular ejection fraction, *LVEDV* left ventricular end-diastolic volume, *LVESV* left ventricular end-systolic volume, *sBP* systolic blood pressure

^a^*p* < 0.001 between G1 and G2

^aa^*p* < 0.01 between G1 and G2

^b^*p* < 0.001 between G3 and G1

^bb^*p* < 0.01 between G3 and G1

^c^*p* < 0.001 between G3 and G2

^cc^*p* < 0.01 between G3 and G1

**Fig. 7:**
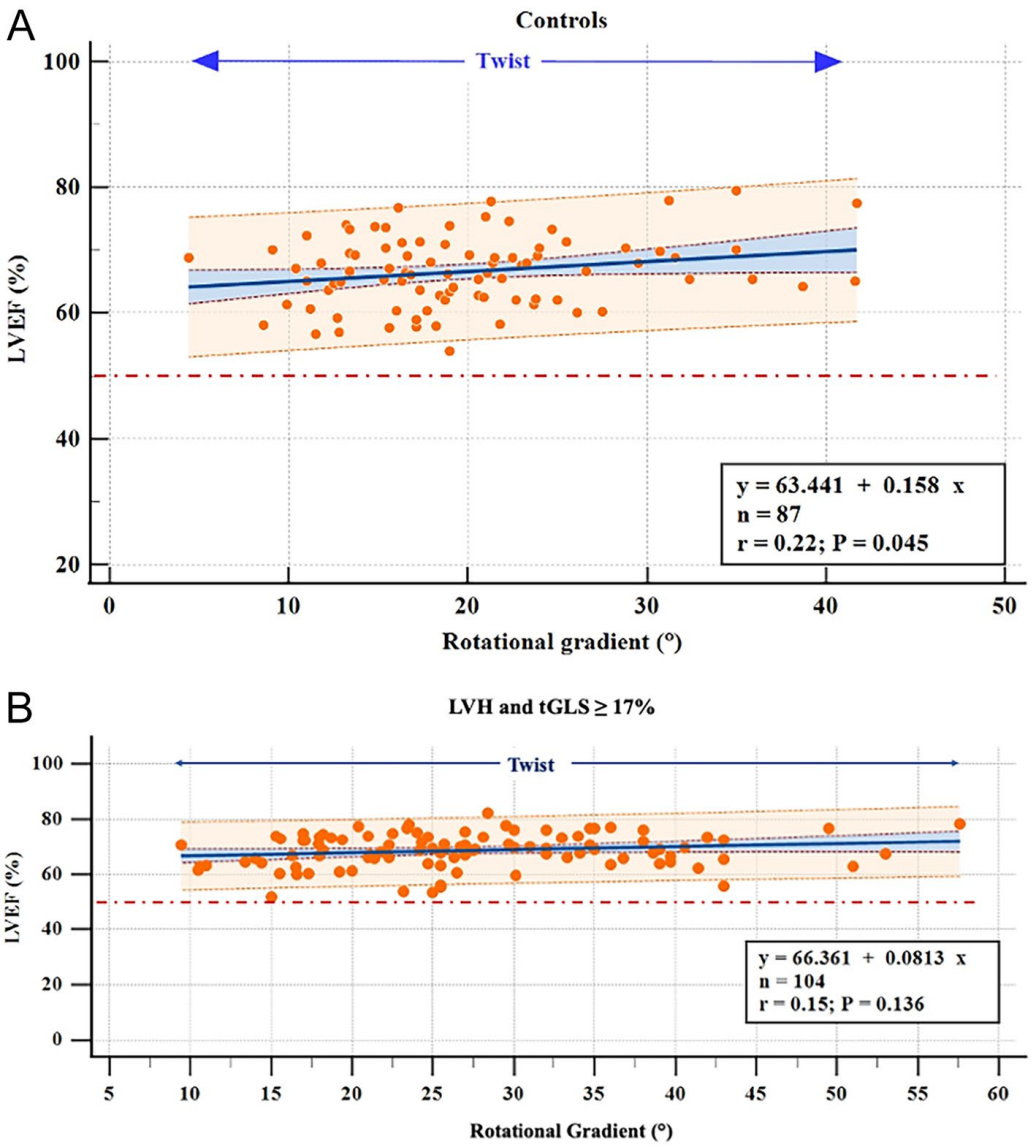
LVEF was $\ge$ 50% in healthy subjects (**A**) and all patients with LVH and normal myocardial function (**B**) (tGLS ≥ 17%, both), so that rotational gradient quantification does not provide additional information about LVEF status. Scatter plot showing the linear relationship between rotational gradient and LVEF in tGLS $\ge$ 17% according to the proposed coding system. It is represented with 5% CI (curves close to the regression line) and 95% prediction

A twist pattern was observed in all healthy controls (Fig. 7A) and in 104 patients with LVH and tGLS ≥ 17% (Fig. 7B). LVEF was $\ge$ 50% in all these subjects, so that normal tGLS $\ge$ 17% indicated normal LVEF. In this context of normal myocardial function, rotational gradient quantification does not provide additional information about LVEF status.

**Fig. 8:**
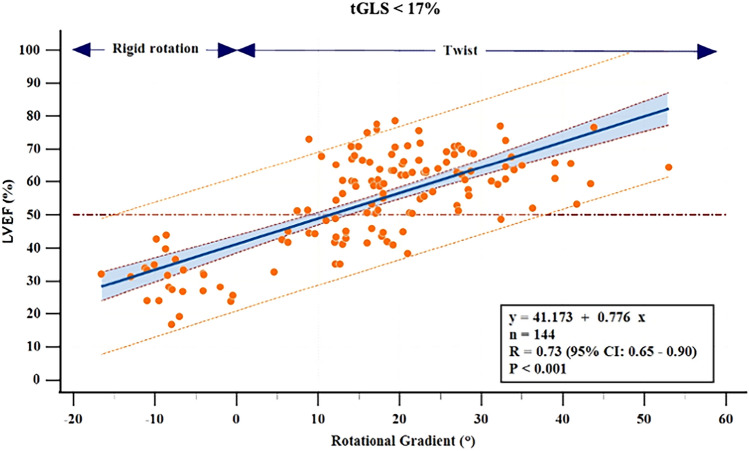
The rotational gradient values reflected a good relation with LVEF and provided useful information about LVEF in subjects with myocardial dysfunction (tGLS < 17%). Scatter plot showing the linear relationship between rotational gradient and LVEF in tGLS < 17% according to the proposed coding system. It is represented with 5% CI (curves close to the regression line) and 95% prediction

Among the 144 patients with tGLS < 17% (myocardial dysfunction), all of whom presented LVH, 34.7% presented LVEF $<$ 50%. Thus, tGLS < 17% can be observed in patients with reduced or preserved LVEF, which means abnormal tGLS < 17% is not an indication of the status of LVEF (Fig. 8). In these patients, twist pattern was present in 120 (83.3%) cases, rigid rotation in 21 (14.6%), and semi-rigid rotation in 3 (2.1%). All patients with rigid or semi-rigid rotation pattern presented LVEF < 50%. Figure 8 is a graphical representation of the rotational gradient values assigned by our coding system to these 144 patients with tGLS < 17%. The rotational gradient values reflected a good relation with LVEF (*r* = 0.73; *p* < 0.001) and provided useful information about LVEF in these subjects with myocardial dysfunction.

Twenty-four patients with rigid or semi-rigid rotation were negatively coded according to our system, and all presented LVEF < 50%. Twenty-two of them were misinterpreted positively (as if there were twist) by the current codification model. Only two patients with clockwise rigid rotation were coded negatively by both coding systems, as clockwise basal rotation was higher than clockwise apical rotation. The current coding system did not distinguish twist from rigid rotation.

The echocardiographic results were reviewed by two observers.

### Intraobserver and Interobserver Reproducibility

The intraclass correlation coefficient was calculated in a random sample of 10 patients, in whom masking and measurements were performed at different times in order to evaluate intra- and interobserver reproducibility of the results. The Bland–Altman method was used for graphic representation of the findings, and the 95% limits of agreement were calculated to obtain the mean. Normal data distribution was confirmed using the Shapiro–Wilk test (Table 2).

**Table 2:** Intraobserver and interobserver variability

Intraclass correlation coefficient (95% CI)		*p*	Mean of difference (95% LOA)
Intraobserver			
tGlobal longitudinal strain	0.86 (0.53-0,96)	< 0.001	− 0.14 (95% LOA: − 2.51 to 2.23)
Twist	0.94 (0.65-0.98)	< 0.001	− 1.15 (95% LOA: − 3.81 to 1.51)
Interobserver			
tGlobal longitudinal strain	0.87 (0.58-0.96)	< 0.001	0.50 (95% LOA: − 1.77 to 2.77)
Twist	0.85 (0.53-0.96)	< 0.001	− 1.13 (95% LOA: − 6.69 to 4.43)

*LOA* limits of agreement

## Discussion

“Measure what is measurable, and make measurable what is not so”, said Galileo. Perhaps one could add “…and code it appropriately”.

Unlike the current coding system, the codification of rotational mechanics that we describe herein makes possible to qualitatively distinguish ventricular twist from rigid rotation and to quantitatively calculate its impact on LVEF.

In a healthy heart, there is longitudinal ventricular shortening of a fundamentally subendocardial origin during systole. Simultaneously, the base and apex rotate in opposite directions to produce ventricular twist, a movement that originates at the transmural and subepicardial levels.

The importance of twist for LVEF has been demonstrated mathematically and experimentally. Assuming the shape of the LV to be an ellipsoid of revolution, Sallin [18] mathematically described how the greatest volume of ventricular ejection is obtained when the myocardial fibers are arranged obliquely along the ventricular wall; in contrast, when they are arranged in a circular fashion, twist does not occur and the ejection volume is smaller. Experimental models have confirmed this to be true [19–21]. On the other hand, absence of twist (rigid rotation) has been related to LVEF < 50% at clinic level [22–25].

### Coding System

If twist values and the absence of twist (rigid rotation) do indeed represent different LVEF values, their correct codification is vital to clinical practice. However, the system currently used to codify rotational gradient, and which is incorporated into available echocardiography devices, does not distinguish twist from rigid rotation. This coding system wrongly assumes that all rotational models indicate the existence of twist, which is calculated through algebraic subtraction by the formula Twist° = (apical rotation)° − (basal rotation)°. Thus, situations as disparate as the presence and absence of twist (rigid rotation, in the latter case) are coded indistinctly as positive or negative, which does not allow them to be differentiated and leads to confusion (Figs. 3 column A, 4 column A and 5 column A, white line). We propose a new and simple system with which to codify the different rotational models of the LV (see Algorithm 1 in “Methods” section) in which twist is represented as a positive value (Fig. 3 column B, broken yellow line) due to its beneficial effect on LVEF, while the absence of twist—and, hence, the presence of rigid or semi-rigid rotation—is represented as a negative value (Figs. 4 column B and 5 column B, broken yellow line) due to its deleterious effect.

As one can observe in Fig. 6A–F, twist and rigid rotation values are represented indistinctly as positive or negative by the current coding system (white line), which does not distinguish one from the other. In the examples cited, the presence of twist would be coded as positive and the presence of rigid rotation as negative if we were to apply our coding system (broken yellow line), thus allowing twist to be distinguished from rigid rotation. It is important to stress that coding of the direction of “rotation” (clockwise vs counterclockwise) should not be confused with coding of the “rotational gradient” (twist-rotational gradient positive vs rigid rotation-rotational gradient negative).

### Relevance of the Coding System in Clinical Practice

Once a coding system has been established, it is important to confirm its relevance in clinical practice. As we observed in our study, normal tGLS indicates a preserved LVEF (Fig. 7A, B). Most heart diseases are characterized by myocardial dysfunction in their initial phases due to fundamentally subendocardial involvement, which is indicated by a reduction in tGLS, even in subclinical stages. In this way, a decrease in tGLS is a sign of myocardial dysfunction. However, as we observed in our patients, it does not determine LVEF, as low tGLS values appear in patients with preserved or reduced LVEF. Therefore, a low tGLS does not provide information about LVEF status. As the disease progresses, transmural and subepicardial function begins to deteriorate. This results in a progressive reduction in twist, until it disappears and is substituted by a rigid rotation pattern, after which there is a severe decline in LVEF [22–25]. In these circumstances, the study of rotational pattern is important, and a coding system needs to be able to differentiate myocardial rotational events if it is to provide additional information about LVEF aside from that provided by measuring tGLS.

In our study, all subjects with normal myocardial function (tGLS ≥ 17%) presented twist (Fig. 7A, B); thus, in a context of normal subendocardial and transmural heart function, rotational gradient quantification does not provide additional information about LVEF status.

However, the situation changes when subendocardial function is abnormal and there is a decrease in tGLS (< 17%). This means the behavior of the transmural function and the pattern of its rotational mechanics, and especially the presence of different degrees of twist or rigid rotation, are all of relevance to LVEF. When we applied our coding system to patients with myocardial dysfunction (tGLS < 17%), progressively lower rotational gradient values were related to lower values of LVEF (*r* = 0.73, *p* < 0.001) (Fig. 8). When twist disappears it is substituted by a rigid rotation pattern whose rotational gradient is negative, which is then followed by a severe decline in LVEF. We noted that, unlike what we observed in subjects with normal myocardial function, both pattern and rotational gradient were indicators of LVEF status in those with myocardial dysfunction.

The current coding system does not distinguish twist from rigid rotation, and the information it provides about LVEF is unreliable. This probably explains why data on rotational mechanics and LVEF are scarce and why the literature on the subject is contradictory.

The information provided by observational or experimental data requires an adequate codification that allows events with different meanings to be distinguished, as in the case of LV rotation pattern. The alternative coding system we propose allows the different ventricular rotational models to be clearly identified, and the rotational gradient values it provides in patients with myocardial dysfunction have a relationship with LVEF.

The LVEF is the end result of all the elements involved in the contraction of the ventricular cavity, the most important part of which is the myocardium. The relevance of the present study lies in the correct codification of indicators other than LVEF in a routine evaluation of LV function. Our data have been obtained in a cross-sectional specific population of healthy subjects and patients with LVH; whether impaired rotational gradient is an early detector of incipient ventricular dysfunction in LVH and other pathologies remains to be seen.

The coding system we propose herein can be applied to any cardiac imaging modality with a simple modification of the device’s software. We have found that it allowed us to qualitatively and quantitatively relate rotational model with LVEF. Along with LVEF, which has enormous clinical value, and the information provided by tGLS, an accurate codification of rotational cardiac mechanics is fundamental for the early identification and stratification of patients at risk of heart failure.

### Limitations

Despite the great advantages offered by 2D-STE, it has several limitations. Its usefulness depends on the quality of the image obtained and it can be inaccurate due to planar movement and the degree of obliquity of the transverse planes. Some of these limitations are overcome with 3D-STE, but a lower temporal resolution, greater susceptibility to image quality in the grey scale, and lack of experience of technical staff continue to represent challenges. It is expected that three-dimensional ultrasound will facilitate the standardization of reference points so that transverse planes can be obtained in an automated way. Our results are based on deformation parameters calculated by one vendor, and intervendor variability may be a factor to be taken into consideration when interpreting the strain values reported herein.

## Abbreviations

LV: Left ventricular
tGLS: Transmural global longitudinal strain
tGCS: Transmural global circumferential strain
LVEF: Left ventricular ejection fraction
LVH: Left ventricular hypertrophy
STE: Speckle tracking echocardiography
